# Folate, Vitamin B12, and Homocysteine Levels in Women With Neural Tube Defect-Affected Pregnancy in Addis Ababa, Ethiopia

**DOI:** 10.3389/fnut.2022.873900

**Published:** 2022-04-08

**Authors:** Winner Kucha, Daniel Seifu, Abenezer Tirsit, Mahlet Yigeremu, Markos Abebe, Dawit Hailu, Dareskedar Tsehay, Solomon Genet

**Affiliations:** ^1^Department of Biochemistry, School of Medicine, College of Health Sciences, Addis Ababa University, Addis Ababa, Ethiopia; ^2^Biochemistry Division of Basic Sciences, University of Global Health Equity, Kigali, Rwanda; ^3^Neurosurgery Unit, School of Medicine, College of Health Science, Addis Ababa University, Addis Ababa, Ethiopia; ^4^Department of Obstetrics and Gynecology, School of Medicine, College of Health Sciences, Addis Ababa University, Addis Ababa, Ethiopia; ^5^Armauer Hansen Research Institute, Addis Ababa, Ethiopia

**Keywords:** NTDs, folate, vitamin B12, homocysteine, ELISA

## Abstract

**Background:**

Neural tube defects (NTDs) are prevalent congenital defects associated with pre-pregnancy diet with low levels of maternal folate. They are linked to severe morbidity, disability, and mortality, as well as psychological and economic burdens.

**Objective:**

The goal of this study was to determine the levels of folate, vitamin B12, and homocysteine in the blood of women who had a pregnancy impacted by NTDs.

**Subjects and Methods:**

A hospital-based case–control study was undertaken between September 2019 and August 2020. The study comprised a total of 100 cases and 167 controls. Enzyme-linked immunosorbent assay (ELISA) was used to determine the levels of folate, vitamin B12, and homocysteine in the serum.

**Results:**

Only 39% of the cases and 54.5% of control mothers reported periconceptional use of folic acid/multivitamin, which indicated a statistically significant difference (*p* = 0.014). Logistic regression indicated that periconceptional use of folic acid/multivitamin was associated with NTDs (*p* = 0.015, OR = 1.873, 95% CI: 1.131–3.101). We found that 57% of the cases and 33.5% of controls, as well as 43% of cases and 20.4% of controls had serum folate and vitamin B12 levels below the cut-off value, respectively. Twenty-seven percent of the cases and 6.6% of controls had hyperhomocysteinemia (HHcy). The median concentrations of folate, vitamin B12, and homocysteine in cases and controls were 4.78 and 8.86 ng/ml; 266.23 and 455 pg/ml; 13.43 and 9.7 μmol/l, respectively. The median concentration of folate (*p* < 0.001) and vitamin B12 (*p* < 0.001) were significantly lower in the cases than controls, while the homocysteine concentration (*p* < 0.001) was significantly lower in the controls than cases. Folate [OR (95% CI) = 1.652 (1.226–2.225; *p* = 0.001)], vitamin B12 [OR (95% CI) = 1.890 (1.393–2.565; *p* < 0.001], and homocysteine [OR (95% CI) = 0.191 (0.09–0.405; *p* < 0.001)] levels were associated with NTDs.

**Conclusion:**

Folate and vitamin B12 are deficient in both cases and control mothers. The lower levels of folate and vitamin B12 with an elevated homocysteine level in NTD-affected pregnancy may be an indication that these biochemical variables were risk factors for NTDs. Folate/multivitamin supplementation and/or food fortification should be promoted.

## Introduction

Vitamins and minerals are crucial components of a diet that are needed in modest amounts to sustain nearly all metabolic activities, such as cell signaling, motility, proliferation, differentiation, and cell death, which govern tissue development, function, and homeostasis. Early in life, these key biological functions allow the fetus to grow and evolve into a healthy offspring. Vitamins and minerals assist a healthy pregnancy at every stage of maternal, placental, and fetal contact (1).

Neural tube defects (NTDs) are congenital abnormalities of the brain and spinal cord caused by a defect in the neural tube closure during embryonic development (2). They are the most common known defects associated with pre-pregnancy nutrition with low levels of maternal folate (3); resulting in significant mortality, morbidity, and disability along with psychological and economic burdens (4).

The etiologies of NTDs are intricate and multifactorial where genetic, life style, and environmental factors seem to take part. Despite their public health impact, knowledge about the etiology of NTDs in humans is insufficient (5). Mothers who are deficient in folate are more likely to produce infants who develop NTDs (6, 7). However, the precise mechanisms involved in the development of NTDs are unclear, although previous studies show a reduced risk of these disorders following folate supplementation (8).

Although vital throughout a lifetime, folate is especially important during embryogenesis. Gestation has been documented as a period when the needs for folate are increased to maintain the requirement for prompt cellular duplication and development of tissues of the mother, the fetus, and placenta, relating to the crucial role it has in the synthesis of protein, DNA, and RNA. Sustaining a sufficient folate level throughout gestational period is essential for the mother's wellbeing as well as for the developing embryo because folate deficiency in pregnancy has been linked with adverse outcomes, including folate-responsive NTDs, disorders of the neural crest, fetal developmental delay, premature birth, low birth weight, and folate deficiency in the newborn (9).

It has been estimated that 50–60% of the NTDs can be avoided by attaining and keeping sufficient folate status pre-conceptionally and within the first few weeks of pregnancy (10). Prevalence estimates of NTDs are high all over the world, with roughly 80% of reported prevalence estimates exceeding 6.0 per 10,000 births (that is the estimated amount that should be achieved through sufficient folic acid consumption peri-conceptionally) (11).

Neural tube defects are widespread among children in Ethiopia with an estimated pooled prevalence of 63.3 cases per 10,000 children. The pooled prevalence of spinal bifida, anencephaly, and encephalocele was 41.09, 18.90, and 1.07 per 10,000 children, respectively (12). Spina bifida and anencephaly are responsible for a large number of stillbirths and infant deaths (13). Despite overwhelming evidence that folic acid lowers the incidence of NTDs, Ethiopia does not currently have a required food fortification program (14).

Folic acid is not the only nutrient that, when deficient, has been linked with an elevated risk of NTDs. Vitamin B-12 insufficiency has been associated with adverse consequences in both mothers and babies including growth defects, spontaneous abortions, preeclampsia, and low birth weight. Given its critical function in neural myelination, brain development, and growth, appropriate vitamin B-12 level peri-conceptionally as well as during gestational period cannot be overstated. Neural tube closure abnormalities may be more common in babies delivered to vitamin B-12 deficient mothers (15).

Homocysteine is an amino acid produced from methionine. Abnormalities in homocysteine metabolism lead to hyperhomocysteinemia (HHcy). HHcy is linked to an increased risk of a variety of diseases, including birth abnormalities. Several studies have found that elevated maternal homocysteine levels are linked to an elevated risk of NTD in the fetus in various populations (16).

## Materials and Methods

### Ethics Approval Statement

The study was approved by Departmental Research Ethics Review Committee of the Department of Biochemistry (Ref. No. SOM/BCHM/098/2010) and Institutional Review Board (IRB) of the College of Health Sciences (CHS), Addis Ababa University (AAU) (Protocol number: 049/18/Biochem). Approval was also obtained from the National Research Ethics Review Committee in the Ministry of Science and Higher Education, Ethiopia (Ref. No. MoSHE/RD/14.1/465/19) and IRB of the Health Bureau of the City Government of Addis Ababa (Ref. No. AAHB/223/227). Permission was also obtained from Tikur Anbessa Specialized Hospital, Zewditu Memorial Hospital, and Gandhi Memorial Hospital. The study's goal was briefly described to the participants who were also told that their responses would be kept strictly confidential. Following the study participants' consent, samples and data were gathered.

### Subjects

The cases were pregnant women ≥18 years old, with a prenatal diagnosis of fetal NTD on prenatal ultrasound. The control groups were gestational age-matched pregnant women, with current pregnancy unaffected by NTD. Both cases and controls were selected from three hospitals (Tikur Anbessa Specialized Hospital, Zewditu Memorial Hospital, and Gandhi Memorial Hospital in Addis Ababa), Ethiopia between September 2019 and August 2020. These hospitals were selected because they are the major teaching hospitals for Addis Ababa University and they provide 24-h obstetrics and gynecology services for the city and surrounding areas. One hundred cases and 167 controls were included in the study. Pregnant mothers with stillbirths or elective termination before the blood draw were not included in the study.

### Data Collection

The study participants were interviewed face to face by trained nurses with a questionnaire, and medical records were reviewed to collect socio-demographic, pregnancy, and disease conditions of the study participants. The questionnaire was pretested on 30 pregnant women in Tekle Haimanot Health Center in Addis Ababa. Body mass index (BMI) was computed by dividing body weight (in kg) in light clothing and without shoes by height (in meter) squared.

### Biochemical Analysis

Four milliliters of venous blood was collected by venipuncture after an overnight fast of at least 8 h from the arm using a syringe and transferred into serum separator tubes, allowed to form a clot by letting it stand in an upright position for at least 30 min, subjected to centrifugation (15 min, 4,000 rpm) at room temperature, divided into aliquots in labeled cryotubes, and transported from the site of collection on an ice box containing frozen ice packs to the core laboratory of the CHS, AAU and stored at −80°C until biochemical analyses. Each serum sample was stored for a maximum of 2 months before analyses.

A competitive enzyme-linked immunosorbent assay (ELISA) (Cloud-Clone Corp. Katy, TX, USA) was used to determine the serum levels of folate, vitamin B12, and homocysteine. The analyses were performed according to the manufacturer's instructions. Each analysis was performed in duplicates and the average of the duplicate measurements were taken. The analyses were done at the immunology laboratory in Armauer Hansen Research Institute, Addis Ababa, Ethiopia.

### Statistical Analysis

Statistical analyses were performed using SPSS for windows program version 25.0 (SPSS Inc., Chicago, IL, USA). Differences in categorical variables between cases and controls were analyzed by the *x*^2^ or the Fisher's exact test where appropriate. Shapiro-Wilk test was used to check for the normality of distribution of folate, vitamin B12, and homocysteine in both cases and controls. Folate, vitamin B12, and homocysteine were not normally distributed and remained non-normal even after log transformation, and hence that non-parametric analysis was performed. Differences between medians were analyzed by Mann–Whitney U test. Logistic regression was used to determine the association of folate, vitamin B12, and homocysteine with NTD-affected pregnancy. Statistical significance was set at *p* < 0.05.

## Results

Among the total of 100 NTDs affected pregnancies, there were 79 spina bifida cases, 17 anencephaly cases, and 4 encephalocele cases.

### Socio-Demographic Characteristics of the Study Participants

The mean age of the cases was 26.8 ± 5.25 while the controls were 27.2 ± 4 years. Most of the cases (46%) were in the age group of 20–24 years, whereas the majority of the controls (38.92%) belong to the age group 25–29 years.

There was no difference in BMI of cases and controls. Ninety-eight percent of the cases and 97% of the controls have a normal BMI (18.5–24.9 kg/m^2^). Eighty-seven percent of cases and 100% of controls were urban dwellers.

In terms of education, 81% of cases and ~96% of controls have primary and above primary-level of education. Almost all of the cases (99%) and more than 96% of the controls were married. Sixty-four percent of the cases and 58.7% of controls have a family size of 3 or less. With respect to religion, Orthodox Christians constitute 62% of the cases and 64% of the controls. The majority of the cases (72%) and controls (55%) were unemployed/housewives. All of cases and 99.4% controls had no history of periconceptional tobacco and alcohol use (Table 1).

**Table 1:** Socio-demographic characteristic of the study participants in three selected hospitals in Addis Ababa, Ethiopia, between September 2019 and August 2020.

**Variables**		**Cases (%)**	**Controls (%)**	***X* ^2^**	***P***
Age (years)	<20 20–24 25–29 30–34 35–39 ≥40	0 46 (46) 39 (39) 11 (11) 2 (2) 2 (2)	1 (0.6) 51 (30.54) 65 (38.92) 38 (22.75) 10 (5.99) 2 (1.2)	11.94	0.021
BMI (kg/m^2^)	<18.5 18.5–24.9 25–29.9 ≥30	1 (1) 98 (98) 1 (1) 0	0 162 (97) 3 (1.8) 2 (1.2)	2.62	0.501
Education	No formal education Primary (1–8) Secondary (9–10/12) Above Secondary	19 (19) 35 (35) 26 (26) 20 (20)	21 (12.57) 59 (35.33) 50 (29.94) 37 (22.16)	2.203	0.531
Marital status	Married/living together Never- married/never lived together Widowed/Divorced/separated	99 (99) 1 (1) 0	161 (96.4) 3 (1.8) 3 (1.8)	1.614	0.593
Family size	≤ 3 4–6 >6	64 (64) 28 (28) 8 (8)	98 (58.68) 62 (37.13) 7 (4.19)	3.452	0.178
Religion	Orthodox Islam Protestant Other	62 (62) 22 (22) 16 (16) 0	107 (64.07) 36 (21.56) 22 (13.17) 2 (1.2)		
Occupation	Employed Private work Daily Laborer Unemployed/House wife	21 (21) 5 (5) 2 (2) 72 (72)	63 (37.72) 12 (7.19) 0 92 (55.09)	11.844	0.005
Tobacco use	Yes No	0 100 (100)	1 (0.6) 166 (99.4)		
Alcohol consumption	Yes No	0 100 (100)	1 (0.6) 166 (99.4)		

### Pregnancy and Disease Conditions

Most of the cases (99%) and the controls (98.8%) are in their second and third trimester of the index pregnancy. Majority of the cases (91%) and the controls (87%) had pregnancies ≤ 3. Periconceptional folic acid/multivitamin supplementation was taken by only 39% of the cases and 54.5% of the controls. A statistically significant difference (*x*^2^ = 6.008, *p* = 0.014) was observed on the periconceptional use folic acid/multivitamin supplementation between cases and controls. Logistic regression analysis indicated the periconceptional use of folic acid/multivitamin to be associated with NTDs (*p* = 0.015, OR = 1.873, 95% CI: 1.131–3.101).

Diabetes was reported in 4% of the cases and 2% of the controls. None of the cases as well as the controls reported a history of use of anticonvulsant medications and treatment with drugs such as isotretinoin/methotrexate periconceptionally (Table 2).

**Table 2:** Pregnancy and disease conditions of the study participants in three selected hospitals in Addis Ababa, Ethiopia, between September 2019 and August 2020.

**Variables**		**Cases (%)**	**Controls (%)**	***X* ^2^**	***p***
Gravidity	≤ 3 >3	91 (91) 9 (9)	145 (86.83) 22 (13.17)	1.062	0.303
Parity	≤ 3 >3	92 (92) 8 (8)	145 (86.83) 22 (13.17)	1.679	0.195
Trimester of pregnancy during diagnosis	First Second Third	1 (1) 61 (61) 38 (38)	2 (1.2) 103 (61.67) 62 (37.13)	0.186	0.954
Periconceptional Folic acid/multivitamin use	Yes No	39 (39) 61 (61)	91 (54.49) 76 (45.51)	6.008	0.014
History of diabetes mellitus	Yes No	4 (4) 96 (96)	3 (1.8) 164 (98.2)	1.190	0.430
Use of any anticonvulsant medications periconceptionally	Yes No	0 100 (100)	0 167 (100)		
Treatment with isotretinoin/methotrexate periconceptionally	Yes No	0 100 (100)	0 167 (100)		

### Serum Folic Acid, Vitamin B12, and Homocysteine Levels

Folate level is classified as <3 ng/ml deficiency, 3–5.99 ng/ml possible deficiency, 6–20 ng/ml normal range (17). Vitamin B12 level is classified as <150 pg/ml severe deficiency, 150–200 pg/ml moderate deficiency, and >201 pg/ml normal (18). Levels of homocysteine <12 μmol/l are considered as optimal, 12–15 μmol/l as borderline, and levels >15–30 μmol/l are defined as moderate HHcy (19). For this study, we used homocysteine <15 μmol/l as normal and >15 μmol/l as HHcy.

Folate, vitamin B12, and homocysteine are not normally distributed even after log transformation in both cases and controls (*p* < 0.001 in all analyses).

The mean ± SD folate level in cases (*n* = 100) was 4.4 ± 1.75 ng/ml, ranging from 1.91 to 6.47 ng/ml, with a median of 4.78 ng/ml (IQR, 2.47–6.14 ng/ml). The mean ± SD folate level in controls (*n* = 167) was 8.01 ± 3.1 ng/ml, ranging from 2.8 to 10.81 ng/ml, with a median of 8.86 ng/ml (IQR, 5.4–10.74 ng/ml). The median folate level was statistically different between cases and controls (*p* < 0.001). Folate level is associated with NTDs (*p* = 0.001), OR (95% CI) = 1.652 (1.226–2.225; Table 3).

**Table 3:** Serum folate, vitamin B12, and homocysteine levels of the study participants in three selected hospitals in Addis Ababa, Ethiopia between September 2019 and August 2020.

**Biochemical** **parameters**	**Median**		**OR (95% CI)**	***p***
	**Cases**	**Controls**		
Folate (ng/mL)	4.78	8.86	1.652 (1.226–2.225)	<0.001
Vitamin B12 (pg/mL)	266.23	455	1.890 (1.393–2.565)	<0.001
Homocysteine (μmol/L)	13.43	9.7	0.191 (0.09–0.405)	<0.001

The mean ± SD vitamin B12 level in cases (*n* = 100) was 292.34 ± 162.5 pg/ml ranging from 91 to 571.75 pg/ml, with a median of 266.23 pg/ml (IQR, 139.17–447.37 pg/ml). The mean ± SD vitamin B12 level in controls (*n* = 167) was 408.26 ± 178.91 pg/ml, ranging from 92.5 to 634 pg/ml, with a median of 455 pg/ml (IQR, 254–585 pg/ml). The median vitamin B12 level was statistically different between cases and controls (*p* < 0.001). Vitamin B12 level is associated with NTDs [*p* < 0.001, OR (95% CI) = 1.890 (1.393–2.565)] (Table 3).

The mean ± SD homocysteine level in cases (*n* = 100) was 13.63 ± 4.2 μmol/l, ranging from 6.4 to 23.25 μmol/l, with a median of 13.43 μmol/l (IQR, 10.55–15.56 μmol/l). The mean ± SD homocysteine level in controls (*n* = 167) was 10.88 ± 3.45 μmol/l, ranging from 6.8 to 23.5 μmol/l, with a median of 9.7 μmol/l (IQR, 8.3–13.2 μmol/l). The median homocysteine level was statistically different between the cases and controls (*p* < 0.001). Homocysteine level is associated with NTDs [*p* < 0.001, OR (95% CI) = 0.191 (0.09–0.405)] (Table 3).

## Discussion

We measured levels of folate, vitamin B12, and homocysteine in the serum to determine the level and examine the association of these biochemical variables with the risk of NTD-affected pregnancy in pregnant women in Addis Ababa, Ethiopia.

Despite the recommendation and advice on supplementing folic acid for all women trying for pregnancy, 61% of the cases and 45.5% of the controls reported no usage of folic acid/multivitamin supplementation peri-conceptionally. This result is statistically significant (*p* = 0.01), which might be one factor for the occurrence of NTDs in the case groups. Previous studies indicated the evidence of reduced NTD occurrence ranging between 35 and 75%, related with the consumption of multivitamins containing folic acid and food with high folate content (20–23).

We found that 57% of the cases and 33.5% of the controls had serum folate levels below 6 ng/ml, which is a folate deficiency state. Furthermore, the median concentration of serum folate was significantly lower in cases than controls (*p* = 0.001). Folate level is associated with NTD-affected pregnancy [OR (95% CI) = 1.652 (1.226–2.225; *p* = 0.001)]. Our result is similar with previous reports (24, 25), which found significantly lower folate levels in cases than controls and a significant link between maternal folate status and the risk of NTDs in the newborn.

Folate status may become a concern under the circumstances of therapeutic drug use such as anticonvulsants (phenytoin, primidone), sulfasalazine, triamterene, and metformin. However, none of our study participants reported the usage of any type of drugs during the periconceptional period.

Folate is essential for proper growth of the fetal brain, skull, and spine, particularly during the first months of pregnancy. Women who do not take the appropriate amount of folic acid supplement, are on restricted diets, have a lower socioeconomic status, or have limited access to nutritionally adequate and safe food are at risk of low folate status (26).

Despite the substantial epidemiological and experimental evidence that links folate level to the risk, occurrence, and recurrence of NTDs (27), the means by which folate helps closure of the neural tube and decreases NTD risk are not fully understood. Since folate serves as an important cofactor for the *de novo* synthesis of purine and thymidine nucleotides and homocysteine remethylation to methionine, it has been proposed that folate may impact NTD risk by affecting the synthesis of nucleotide and cell replication, increasing homocysteine, or impairing cellular methylation potential and gene expression (28). However, it is improbable that one mechanism will serve to explain the association between folate level and risk of NTDs. It is more probable to be a result of the intricate interplay between folate, environmental factors, and genetics (29, 30).

The reduced levels of folate found in our study may be an indication that the majority of the mothers are on deficiency of folate containing foods. Various studies have proposed that inadequate folate consumption was associated to NTDs. Although failed to reach significance, several studies have reported reduced amounts of folates in plasma, red blood cells, or amniotic fluid in women with NTD-affected fetuses or giving birth to NTD-affected offsprings, compared to control pregnant women (31).

Low levels of vitamin B12 in mothers' amniotic fluid and serum have been connected to the risk of NTDs in the fetus. The vitamin B12 levels observed in our study showed that there is a significantly lower level of vitamin B12 in case mothers as compared to the controls (*p* = 0.001). Moreover, 43% of case mothers have vitamin B12 levels below the cut-off value of 200 pg/ml. Vitamin B12 level is associated with NTD-affected pregnancy [OR (95% CI) = 1.890 (1.393–2.565); *p* < 0.001]. Our finding agrees with previous reports of a significantly lower level of vitamin B12 in cases than in control mothers (25, 32). Low vitamin B12 has been shown to be an independent risk factor for NTDs (33). It has been noted that the reports of 24 studies on the association of decreased maternal vitamin B12 status and risk for NTDs make it obvious that decreased maternal vitamin B12 level elevates the risk of NTD to the developing embryo. Additionally, the risk seems to be regardless of maternal folate level (34).

A functional deficiency in folate and vitamin B12 can cause problems with homocysteine metabolism, which can disrupt the synthesis of DNA and transcription, particularly in cellular differentiation during embryogenesis (35). Our result indicated that a significant percentage of case mothers have HHcy as compared to control mothers (27 vs. 6.6%, *p* = 0.001), and the median concentration of homocysteine in case mothers is significantly higher than the control mothers (*p* < 0.001), and homocysteine level is associated with NTD-affected pregnancy [OR (95% CI) = 0.191 (0.09–0.405; *p* < 0.001)]. The hyperhomocysteinemia observed in case mothers may be a result of folate or vitamin B12 metabolic disturbance (36).

Previous studies indicated a significant relationship of elevated maternal plasma homocysteine level with NTDs in the offspring (37, 38). In both animal models and human clinical populations, a link between elevated homocysteine, independent of blood folate levels, and poor pregnancy outcomes has been demonstrated (39–45).

Homocysteine levels were found to be significantly reduced following the combined use of folic acid and vitamin B12. In some regions, the consumption of vitamin B12 and vitamin B6 together with folic acid for the prevention of NTDs and vascular disease has been adopted. The usage of these vitamins augments homocysteine reduction and prevention of NTDs (46).

According to the 2016 Ethiopian national micronutrient survey report, the prevalence of vitamin B12, serum folate, and RBC folate deficiency among non-pregnant women aged 15–49 was 15.1, 17.3, and 32%, respectively (47). This report, together with our finding, may be of value for initiating folic acid interventions in Ethiopia for urgent reductions in NTD cases.

## Conclusion

The significantly reduced level of folate and vitamin B12 status found in cases than control mothers and a tendency for a higher homocysteine concentration in NTD-affected pregnancy supports the view that folate and vitamin B12 deficiency increases the risk of NTD-affected pregnancy. We suggest that sufficient consumption of folate and vitamin B12 containing foods or supplements regularly may help in maintaining adequate folate and vitamin B12 levels periconceptionally and throughout pregnancy to prevent NTDs or for the health benefits of the mother and the fetus. In Ethiopia, until fortification of food with folic acid and vitamin B12 or multivitamin supplementation is incorporated into preconception care, the measurement of serum folate, vitamin B12, or homocysteine may help identify those women at high risk of developing pregnancies affected by NTDs. Folate and vitamin B12 levels were measured after the critical time for neural tube closure and may not be indicative of the levels at that time. Maternal serum alpha-fetoprotein levels were not measured, only ultrasound was used for diagnosis of NTDs. This is the first study in Ethiopia to determine the levels of folate, vitamin B12, and homocysteine in women with NTD-affected pregnancy.

## Data Availability Statement

The original contributions presented in the study are included in the article/supplementary material, further inquiries can be directed to the corresponding authors.

## Ethics Statement

The studies involving human participants were reviewed and approved by National Research Ethics Review Committee at the Ministry of Science and Higher Education—Ethiopia (Ref. No. MoSHE/RD/14.1/465/19). The patients/participants provided their written informed consent to participate in this study.

## Author Contributions

WK, SG, DS, AT, MY, and MA conceived the idea of the presented work and gave final approval of the version to be published. WK, SG, DS, and MA planned the experiments. WK collected samples. WK, DH, and DT carried out laboratory analysis and were involved in the interpretation of data. WK wrote the manuscript with input from all authors. All authors contributed to the article and approved the submitted version.

## Funding

Addis Ababa University supported expenses associated with data and sample collection. Armauer Hansen Research Institute, Addis Ababa, Ethiopia support all expenses related to laboratory analysis.

## Conflict of Interest

The authors declare that the research was conducted in the absence of any commercial or financial relationships that could be construed as a potential conflict of interest.

## Publisher's Note

All claims expressed in this article are solely those of the authors and do not necessarily represent those of their affiliated organizations, or those of the publisher, the editors and the reviewers. Any product that may be evaluated in this article, or claim that may be made by its manufacturer, is not guaranteed or endorsed by the publisher.

## Abbreviations

BMI: body mass index
ELISA: enzyme-linked immunosorbent assay
HHcy: hyperhomocysteinemia
IRB: Institutional Review Board
NTD: neural tube defect.
